# Factors associated with left ventricular diastolic dysfunction in patients with septic shock

**DOI:** 10.1186/s40001-022-00761-5

**Published:** 2022-07-27

**Authors:** Wei-Dong Ge, Feng-Zhi Li, Bang-Chuan Hu, Li-Hong Wang, Ding-Yuan Ren

**Affiliations:** 1 Department of Ultrasonography, Zhejiang Provincial People’s Hospital and People’s Hospital of Hangzhou Medical College, Hangzhou, 310000 Zhejiang China; 2 Present Address: Department of Ultrasonography, The Second Affiliated Hospital of Zhejiang Chinese Medical University, Hangzhou, Zhejiang China

**Keywords:** Sepsis, Septic shock, Left ventricular diastolic dysfunction, Ultrasound, Risk factor

## Abstract

**Purpose:**

To investigate risk factors associated with left ventricular diastolic dysfunction (LVDD) of patients with septic shock.

**Materials and methods:**

Patients with septic shock concomitant with or without LVDD were retrospectively enrolled and divided into the LVDD group (*n* = 17) and control without LVDD (*n* = 85). The clinical and ultrasound data were analyzed.

**Results:**

A significant (*P* < 0.05) difference existed between the two groups in serum creatinine, APACHE II score, serum glucose, triglyceride, BUN, FT4, LAVI, mitral E, average e’, E/average e’, septal e’, septal e’/septal s’, E/septal e’, lateral s’, lateral e’, and E/lateral e’. LAVI > 37 mL/m^2^, septal e’ < 7 cm/s (OR 11.04, 95% CI 3.38–36.05), septal e’/septal s’ < 0.8 (OR 4.09, 95% CI 1.37–12.25), E/septal e’ > 15 (OR 22.86, 95% CI 6.09–85.79), lateral e’ < 8 cm/s (OR 9.16, 95% CI 2.70–31.07), E/lateral e’ > 13 (OR 52, 95% CI 11.99- 225.55), lateral s’ < 10 (OR 3.36, 95% CI 1.13–9.99), average e’ > 10, E/average e’ > 10 (OR 9.53, 95% CI 2.49–36.46), APACHE II score > 16 (OR 3.33, 95% CI 1.00–11.03), SOFA > 5 (or 3.43, 95% CI 1.11–10.60), BUN > 12 mmol/L (OR 3.37, 95% CI 1.15–9.87), serum creatinine > 146* μmol/L* (OR 5.08, 95% CI 1.69–15.23), serum glucose > 8 mmol/L (OR 3.36, 95% CI 1.09–10.40), and triglyceride > 1.8 mmol/L were significant (P < 0.05) risk factors for LVDD. LAVI > 37 ml/m^2^, lateral e’ < 8 cm/s, E/lateral e’ > 13, and SOFA > 5 were significant (P < 0.05) independent risk factors for LVDD. ROC curve analysis demonstrated that the cut-off value and AUC were 37.09 mL/m^2^ and 0.85 for LAVI, 8.00 cm/s and 0.89 for lateral e’, 12.86 and 0.82 for E/lateral e’, and 5.00 and 0.69 for SOFA, respectively.

**Conclusion:**

Left atrial volume index, mitral lateral e’, E/lateral e’, and SOFA score are significant independent risk factors for predicting left ventricular diastolic dysfunction in patients with septic shock.

## Introduction

Sepsis is caused by patients’ exaggerated response to an infection and is related to profound hemodynamic interference, leading to multi-organ failure and even significantly high mortality and morbidity when the initial disease process evolves into the circulatory system [1–5]. Patients with septic shock may experience circulatory, metabolic and cellular interference associated with a high mortality. Septic cardiomyopathy may also occur in septic shock with an incidence of up to 80% [6, 7]. Besides left ventricular (LV) systolic impairment, LV diastolic dysfunction (LVDD) has also been revealed to be a potent predictor of sepsis-related mortality because the hemodynamic-effective cardiac function depends largely on the normal diastolic function of the cardiac muscle [6, 8–10]. LV diastolic function is significantly influenced by hemodynamic conditions, including the heart rate, LV filling time, preload and afterload, and assessment of LV diastolic function is crucial for the diagnosis of cardiac failure and evaluation of the hemodynamic state of patients with acute heart failure [11, 12]. Assessment of hemodynamic alterations in septic shock has revealed that diastolic dysfunction occurs in over 50% of patients with septic shock and that diastolic dysfunction is an independent factor for mortality [10, 13]. At present, LVDD has been used as an independent factor to predict long-term poor prognosis in patients with chronic heart failure, especially in elderly patients with cardiovascular diseases [14, 15]. However, controversy may exist in the prediction value of LVDD in patients with septic shock [8, 9, 16].

Tissue Doppler imaging is used to evaluate LV muscle deformation by measuring the velocity of change in myocardial length, including the systolic (S), early diastolic (e’) and late diastolic velocities (a’). The mitral inflow velocity (E) and late diastolic peak velocity (A) are measured with the pulse wave Doppler. In tissue Doppler measurement, the e’ wave speed and the E/e’ ratio correlated well with the LVDD [17]. It was also found that E/e’ was closely related to pulmonary capillary wedge pressure (PCWP), LV filling pressure and LV mean diastolic pressure in different heart disease states [18]. In patients with diastolic heart failure, some clinical factors and increases of left atrial volume index (LAVI) and brain natriuretic peptide (BNP) in circulation are related to LVDD, and timely intervention through early identification of risk factors for LVDD can improve the clinical prognosis [19, 20]. However, the risk factors for LVDD in patients with severe sepsis and septic shock are unknown, and identification and timely management of these risk factors would help improve the prognosis of patients with severe sepsis and septic shock. This study was consequently performed to investigate risk factors associated with LVDD in patients with severe sepsis and septic shock.

## Materials and methods

### Subjects

This retrospective one-center study was approved by the ethics committee of Zhejiang Provincial People’s Hospital and People’s Hospital of Hangzhou Medical College, and all patients or their family members had provided written informed consent to participate. Between July 2018 and December 2020, patients with septic shock concomitant with or without LVDD in our hospital were enrolled. The inclusion criteria were patients with septic shock concomitant with or without LVDD who had been examined with tissue Doppler imaging. The exclusion criteria were patients with septic shock who died within 24 h after diagnosis, end-stage malignant tumors, severe hepatic and renal dysfunction, concomitant with autoimmune diseases, long-term use of immunosuppressant, and organ transplantation.

### Parameters for evaluation

Clinical data including age, sex, systolic and diastolic pressures, baseline diseases, infection, mechanical ventilation, and mortality were recorded. The acute physiology and chronic health evaluation (APACHE) II score and sequential organ failure assessment (SOFA) score were assessed [21, 22]. As a widely used and well-proven scoring system for evaluating organ dysfunction, the SOFA is also a valid approach to predict in-hospital mortality in patients with suspected infection. The laboratory tests including blood routine, blood gas, hepatic function and thyroid function were measured.

Twenty-four hours after the patient was admitted into the intensive care unit (ICU), echocardiography was performed by an experienced ultrasound physician using the Vivid S6 Doppler echocardiography machine (Vivid S6, GE healthcare, Horten, Norway). The following parameters were measured: LV end-diastolic dimension (LVEDD), LV end-systolic dimension (LVESD), interventricular septum thickness (IVST), left atrial volume index (LAVI), posterior wall thickness (PWT), early diastolic mitral inflow velocity (E), late diastolic peak velocity (A), deceleration time of E-wave (DT), septal e’, lateral e’, septal s’, septal e’/s’, average e’, peak systolic velocity of mitral annulus (S’), E/A, E/lateral e’, E/septal e’, and E/average e’. LV systolic dysfunction was defined as LV ejection fraction (LVEF) < 50%. In the current guidelines [11], the following four variables are considered when determining LVDD in absence of myocardial disease in two-dimensional echocardiography: LV lateral wall E/e’ (average > 14, septal > 15, or lateral > 13), annular e’ velocity (septal e’ < 7 cm/s or lateral e’ < 10 cm/s), LAVI > 34 ml/m^2^, and peak velocity of tricuspid regurgitation (TR) > 2.8 m/s. LVDD is present if over half of the available parameters meet these cut-off values.

On the morning of the second day after admission into the ICU, 2 ml venous blood was collected from every patient and added into an anticoagulation tube containing 0.1 ml 0.2% ethylene diamine tetraacetic acid (EDTA). After the blood was well shaken at room temperature, it was centrifuged at 2000 r/min with a centrifugation radius of 5.5 cm for 10 min to separate the plasma, and the supernatant was absorbed for examination. The immunofluorescence, triage meter plus diagnostic instrument and supporting reagents were used for detection of troponin I (TNI), serum creatinine, procalcitonin, and D-dimer.

Two authors assessed all the data independently. If in disagreement, a third senior author was involved to reach an agreement.

### Statistical analysis

The statistical analysis was performed with the SPSS software (version 19.0, IBM, Chicago, IL, USA). Measurement data were presented as mean ± standard deviation if in normal distribution and tested with the t test but as median and interquartile range if in skew distribution and tested with the Chi-square test. Enumeration data were presented as numbers and percentages and tested with the Chi-square test. The logistic regression analysis was used to predict the risk factors of LVDD. Significant factors in univariate logistic regression analysis were entered for multivariate logistic regression analysis after excluding those factors with collinearity. Receiver operating characteristic (ROC) curve analysis was performed with calculation of the area under the curve (AUC), cut-off value, sensitivity, specificity, positive and negative predictive values. The significant P was set at < 0.05.

## Results

A total of 102 patients in sinus rhythm were enrolled and divided into the LVDD group with LVDD (*n* = 17) and the control (*n* = 85) without LVDD (Table 1). A significant (*P* < 0.05) difference existed between the two groups in serum creatinine (218.61 ± 35.68 μmol / L vs. 132.34 ± 15.96 μmol / L), APACHE II score (19.71 ± 1.37 vs. 15.98 ± 0.61), serum glucose (11.88 ± 0.91 vs. 8.17 ± 0.41 mmol/L), triglyceride (3.12 ± 0.35 vs. 1.51 ± 0.15 mmol/L), BUN (18.85 ± 2.68 vs. 11.39 ± 1.20), FT4 (12.94 ± 0.97 vs. 10.78 ± 0.45 pmol/L), LAVI (64.44 ± 4.37 vs. 37.17 ± 1.95 mL/mm^2^), mitral E (0.95 ± 0.06 vs. 0.81 ± 0.03 m/s), average e’ (6.12 ± 0.73 vs. 10.34 ± 0.33 cm/s), E/average e’ (16.40 ± 1.00 vs. 8.43 ± 0.45), septal e’ (6.00 ± 0.72 vs. 9.41 ± 0.32 cm/s), septal e’/septal s’ (0.74 ± 0.08 vs. 0.99 ± 0.04), E/septal e’ (17.87 ± 1.28 vs. 9.41 ± 0.57), lateral s’ (8.94 ± 0.83 vs. 10.83 ± 0.37 cm/s), lateral e’ (6.24 ± 0.85 vs. 11.27 ± 0.38 cm/s), and E/lateral e’ > 13 (16.22 ± 0.97 vs. 7.82 ± 0.43) (Tables 1, 2, 3). No significant (*P* > 0.05) differences were found in the other parameters (Tables 1, 2, 3), especially those in the hepatic function and thyroid function.

**Table 1:** Data and laboratory analysis

Variables	Septic shock with LVDD (n = 17)	Septic shock without LVDD (n = 85)	P
Female	3 (17.65%)	14 (16.47%)	0.91
Male	14 (82.35%)	71(83.53%)	
Age (y)	68.24 ± 4.27	61.38 ± 1.91	0.15
BMI (kg / m2)	22.89 ± 0.55	22.41 ± 0.25	0.44
Systolic pressure (mmHg)	134.47 ± 4.51	126.34 ± 2.09	0.11
Diastolic pressure (mmHg)	65.41 ± 3.61	69.78 ± 1.68	0.27
Creatinine (μmol / L)	218.61 ± 35.68	132.34 ± 15.96	0.03
APACHE II score	19.71 ± 1.37	15.98 ± 0.61	0.02
SOFA	8.59 ± 0.88	5.85 ± 0.39	0.005
BNP (ng/L)	733.12 ± 178.79	473.23 ± 82.04	0.19
Albumin (g/L)	31.31 ± 1.30	32.08 ± 0.58	0.59
Lactic acid (mmol/L)	2.24 ± 0.40	2.08 ± 0.17	0.71
Troponin (μg/L)	0.18 ± 0.41	0.64 ± 0.17a	0.31
WBC (109/L)	9.97 ± 1.85	12.29 ± 0.82	0.25
Hemoglobin (g/L)	89.41 ± 5.06	93.54 ± 2.26	0.46
Hematocrit (L/L)	27.80 ± 1.53	29.13 ± 0.69	0.43
Platelet (109/L)	170.65 ± 37.97	184.11 ± 16.98	0.75
RDW (%)	15.57 ± 0.46	15.21 ± 0.20	0.48
Serum glucose (mmol/L)	11.88 ± 0.91	8.17 ± 0.41	0.0003
Cholesterol (mmol/L)	3.07 ± 0.34	2.86 ± 0.15	0.56
Triglyceride (mmol/L)	3.12 ± 0.35	1.51 ± 0.15)	< 0.0001
Aspartate aminotransferase (u/L)	189.47 ± 60.48	76.66 ± 27.05	0.09
Alanine aminotransferase (u/L)	76.23 ± 29.66	65.83 ± 13.27	0.75
Total bilirubin (μmol/L)	27.13 ± 25.10	32.31 ± 11.23	0.85
Direct bilirubin (μmol/L)	13.18 ± 3.18	8.61 ± 5.7	0.19
Globulin (g/L)	27.71 ± 1.58	26.59 ± 0.71	0.52
CRP (mg/L)	111.77 ± 24.28	111.33 ± 10.51	0.98

LVDD, left ventricular diastolic dysfunction; BMI, body mass index; BNP, brain natriuretic peptide; WBC, white blood cell; RDW, erythrocyte distribution width; SOFA, sequential organ failure; CRP, C-reactive protein

**Table 2:** Blood gas and thyroid function analysis

	Septic shock with LVDD (n = 24)	Septic shock without LVDD (n = 75)	p
BUN (mmol/L)	18.85 ± *2.*68	11.39 ± *1.20*	0.01
PO_2_ (mmHg)	106.36 ± 9.52	111.46 ± 4.26	0.63
PCO_2_ (mmHg)	38.78 ± 2.70	39.08 ± 1.21	0.92
Blood oxygen saturation (%)	91.01 ± 2.98	95.36 ± 1.33	0.18
Na (mmol/L)	143.06 ± 1.71	141.51 ± 0.77	0.41
K (mmol/L)	3.67 ± 0.14	3.65 ± 0.06	0.91
Cl (mmol/L)	108.12 ± 1.88	108.52 ± 0.84	0.85
Ca (mmol/L)	1.09 ± 0.03	1.12 ± 0.01	0.23
Phosphorus	1.16 ± 0.18	1.08 ± 0.08	0.67
Base excess (mmol/L)	0.60 ± 1.25	0.55 ± 0.56	0.97
T3 (nmol/L)	0.44 ± 0.07	0.42 ± 0.03	0.76
T4 (nmol/L)	61.23 ± 6.13	50.88 ± 2.82	0.13
TSH (mU/L)	0.87 ± 0.90	1.76 ± 0.41	0.38
FT3 (pmol/L)	1.63 ± 0.14	1.73 ± 0.07	0.52
FT4 (pmol/L)	12.94 ± 0.97	10.78 ± 0.45	0.04
TSAb (%)	29.39 ± 50.64	80.69 ± 24.26	0.36

*LVDD* left ventricular diastolic dysfunction, *BUN* blood urea nitrogen

**Table 3:** Ultrasound measurements of cardiac data

	Septic shock with LVDD (n = 24)	Septic shock without LVDD (n = 75)	P
LAVI (mL/m^2^)	64.44 ± 4.37	37.17 ± 1.95	< 0.0001
LVDV (mm^3^)	113.06 ± 8.92	117.71 ± 3.99	0.64
LVSV (mm^3^)	48.29 ± 5.88	45.82 ± 2.62	0.70
LVEDD (mm)	49.47 ± 1.61	49.26 ± 0.72	0.90
LVESD (mm)	32.53 ± 1.64	32.65 ± 0.73	0.95
IVST (mm)	10.06 ± 0.41	9.69 ± 0.18	0.41
PWT (mm)	10.12 ± 0.34	9.80 ± 0.15	0.39
LVEF (min)	54.60 ± 6.32	59.83 ± 2.95	0.46
DT (m/s)	137.21 ± 14.75	142.14 ± 6.25	0.76
Mitral E (m/s)	0.95 ± 0.06	0.81 ± 0.03	0.04
Mitral A (m/s)	0.87 ± 0.07	0.78 ± 0.03	0.26
E/A	1.26 ± 0.13	1.09 ± 0.06	0.25
Average e' (cm/s)	6.12 ± 0.73	10.34 ± 0.33	< 0.001
E/average e’	16.40 ± 1.00	8.43 ± 0.45	< 0.001
Septal s’ (cm/s)	9.06 ± 0.71	9.95 ± 0.32	0.26
Septal e’(cm/s)	6.00 ± 0.72	9.41 ± 0.32	< 0.0001
Septal e’/septal s’	0.74 ± *0.08*	0.99 ± *0.04*	0.009
E/septal e’	17.87 ± 1.28	9.41 ± 0.57	< 0.0001
Lateral s’ (cm/s)	8.94 ± 0.83	10.83 ± 0.37	0.04
Lateral e’ (cm/s)	6.24 ± 0.85	11.27 ± 0.38	< 0.0001
E/lateral e’	16.22 ± 0.97	7.82 ± 0.43	< 0.0001
Septal a’ (cm/s)	10.18 ± 0.84	11.04 ± 0.39	0.35
Lateral a’ (cm/s)	11.06 ± 0.87	11.92 ± 0.40	0.37

*LVDD* left ventricular diastolic dysfunction, *LAVI* left atrial volume index, *LVDV* left ventricular diastolic volume, *LVSV* left ventricular systolic volume, *LVEDD* left ventricular end-diastolic dimension, *LVESD* left ventricular end-systolic dimension; IVST, interventricular septum thickness; PWT, posterior wall thickness, *LVEF* left ventricular ejection fraction, *DT* deceleration time of E-wave, *A* late diastolic peak velocity, *S*’ peak systolic velocity of mitral annulus, *E* early diastolic mitral inflow velocity, *e*’ early diastolic velocity

Univariate logistic regression analysis revealed that LAVI > 37 mL/m^2^, septal e’ < 7 cm/s (OR 11.04, 95% CI 3.38–36.05), septal e’/septal s’ < 0.8 (OR 4.09, 95% CI 1.37–12.25), E/septal e’ > 15 (OR 22.86, 95% CI 6.09–85.79), lateral e’ < 8 cm/s (OR 9.16, 95% CI 2.70–31.07), E/lateral e’ > 13 (OR 52, 95% CI 11.99–225.55), lateral s’ < 10 (OR 3.36, 95% CI 1.13–9.99), average e’ < 7.5 cm/s, E/average e’ > 10 (OR 9.53, 95% CI 2.49–36.46), APACHE II score > 16 (OR 3.33, 95% CI 1.00–11.03), SOFA score > 5 (OR 3.43, 95% CI 1.11–10.60), BUN (blood urea nitrogen) > 12 mmol/L (OR 3.37, 95% CI 1.15–9.87), serum creatinine > 146 μmol*/L* (OR 5.08, 95% CI 1.69–15.23), serum glucose > 8 mmol/L (OR 3.36, 95% CI 1.09–10.40), and triglyceride > 1.8 mmol/L were significant (*P* < 0.05) risk factors for LVDD (Table 4). Multivariate logistic regression analysis using the parameters of LAVI, E/lateral e’, E/septal e’, E/average e’, septal e’/septal s’, lateral e’, septal e’, APACHE II score, serum creatinine, SOFA score, triglyceride, BUN, and serum glucose demonstrated that LAVI > 37 mL/m^2^, E/lateral e’ > 13, SOFA > 5, and lateral e’ < 8 cm/s were significant (*P* < 0.05) independent risk factors for LVDD (Table 4). Significant parameters in the univariate analysis with collinearity were excluded in the multivariate logistic regression analysis.

**Table 4:** Univariate and multivariate logistic regression analysis of patients with septic shock

*Variables*	*Univariate logistic regression*			*Multivariate logistic regression*	
	*OR*	*95% CI*	*p*	*χ2*	*P*
LAVI > 37 mL/m^2^	25.21	3.19–199.17	< 0.0001	51.98	< 0.001
Septal e’ < 7 cm/s	11.04	3.38–36.05	< 0.0001		
Septal e’/septal s’ < 0.8	4.09	1.37–12.25	0.01		
E/septal e’ > 15	22.86	6.09–85.79	< 0.0001		
Lateral e’ < 8 cm/s	9.16	2.70–31.07	< 0.0001		
E/lateral e’ > 13	52	11.99–225.55	< 0.0001	140.25	< 0.0001
Lateral s’ < 10 cm/s	3.36	1.13–9.99	0.02		
Average e' < 7.5 cm/s	14.93	3.89–57.27	< 0.0001	160.07	< 0.0001
E/average e’ > 10	9.53	2.49–36.46	**0.0002**		
APACHE II score > 16	3.33	1.00–11.03	0.04		
SOFA > 5	3.77	1.01–14.10	0.03	11.42	0.0007
BUN > 12 mmol/L	3.37	1.15–9.87	0.03		
Serum creatinine > 146 μmol* / L*	5.16	1.72–15.47	0.003		0.01
Serum glucose > 11 mmol/L	38.56	2.74–26.76	0.0001		0.02
Triglyceride > 1.8 mmol/L	7.19	2.28–22.67	0.0006		

*LAVI* left atrial volume index, *OR* odds ratio, *CI* confidence interval, *BNP* brain natriuretic peptide, *RDW* erythrocyte distribution width, *BUN* blood urea nitrogen

**Fig.1:**
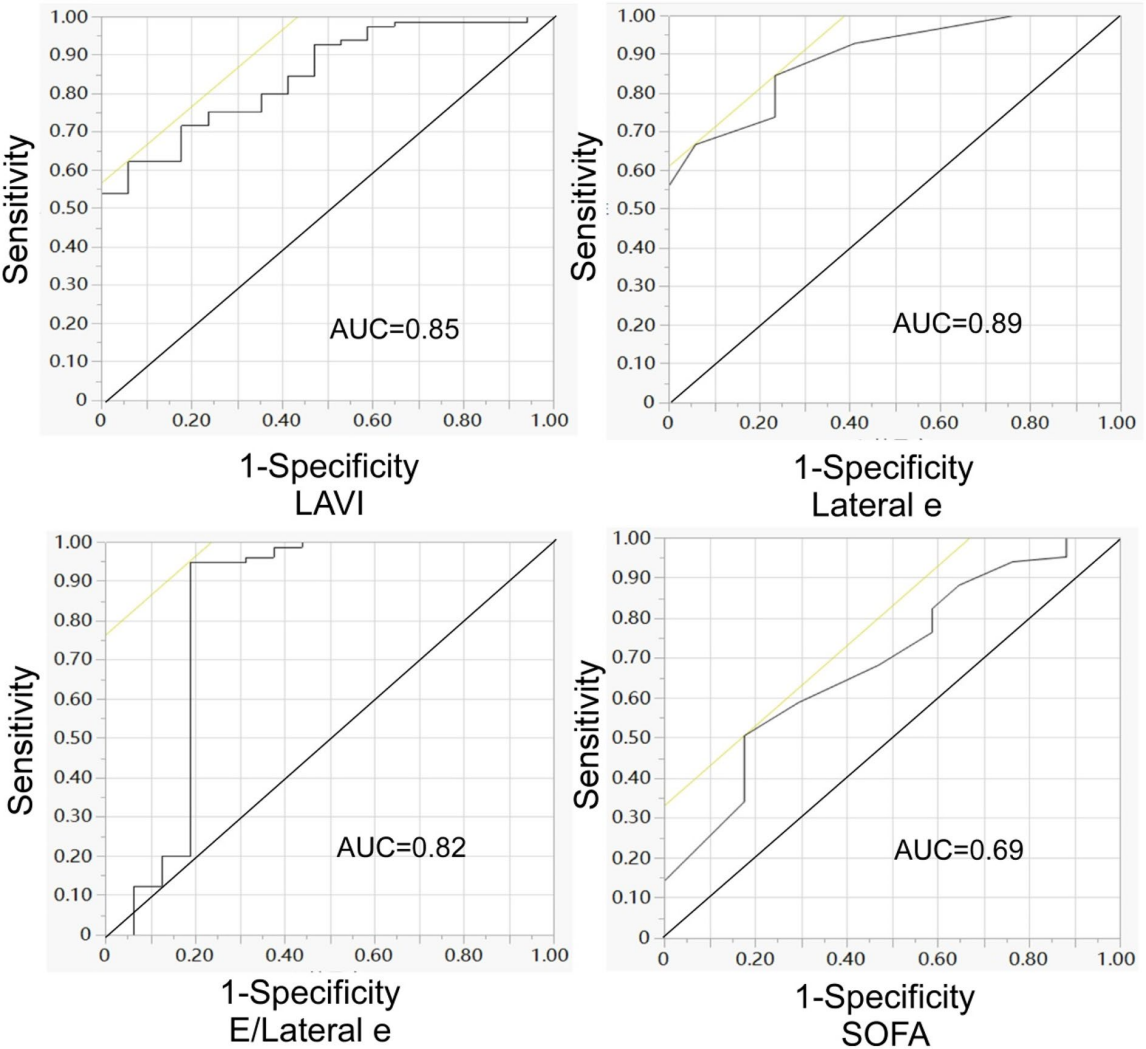
Receiver operating characteristic (ROC) curve analysis of left atrial volume index (LAVI), lateral e’, E/lateral e’, and sequential organ failure (SOFA) as independent risk factors for predicting left ventricular diastolic dysfunction caused by septic shock

ROC curve analysis of independent risk factors in predicting LVDD caused by septic shock demonstrated that the cut-off value and AUC were 37.09 mL/m^2^ and 0.85 for LAVI, 8.00 cm/s and 0.89 for lateral e’, 12.86 and 0.82 for E/lateral e’, and 5.00 and 0.69 for SOFA, respectively (Table 5 and Fig. 1). The sensitivity and specificity were 0.62 and 0.94 for LAVI, 0.85 and 0.76 for lateral e’, 0.95 and 0.81 for E/lateral e’, and 0.51 and 0.82 for SOFA, respectively. The positive and negative predictive values were 0.98 and 0.94 for LAVI, 0.95 and 0.50 for lateral e’, 0.96 and 0.76 for E/lateral e’, and 0.93 and 0.25 for SOFA, respectively.

**Table 5:** ROC curve analysis of independent risk factors for LVDD

Variables	Cut-off value	AUC	Sensitivity	Specificity	Youden index	PPV	NPV
LAVI (mL/m^2^)	37.09	0.85	0.62	0.94	0.56	0.98	0.94
Lateral e’ (cm/s)	8.00	0.89	0.85	0.76	0.61	0.95	0.50
E/lateral e’	12.86	0.82	0.95	0.81	0.76	0.96	0.76
SOFA	5.00	0.69	0.51	0.82	0.33	0.93	0.25

*ROC* receiver operating characteristics, *LVDD* left ventricular diastolic dysfunction, *AUC* area under the ROC curve, *PPV* positive predictive value, *NPV* negative predictive value, *LAVI* left atrial volume index, *SOFA* sequential organ failure

## Discussion

In this study investigating risk factors associated with LVDD in patients with septic shock, it was found that patients with septic shock and combined LVDD are significantly different from those without LVDD in serum creatinine, APACHE II score, serum glucose, triglyceride, BUN, FT4, LAVI, mitral E, average e’, E/average e’, septal e’, septal e’/septal s’, E/septal e’, lateral s’, lateral e’, and E/lateral e’. LAVI > 37 mL/m^2^, septal e’ < 7 cm/s, septal e’/septal s’ < 0.8, E/septal e’ > 15, lateral e’ < 8 cm/s, E/lateral e’ > 13, lateral s’ < 10, average e’ > 10, E/average e’, APACHE II score > 16, SOFA > 5, BUN > 12 mmol/L, serum creatinine > 146* μmol/L*, serum glucose > 8 mmol/L, and triglyceride > 1.8 mmol/L are significant (*P* < 0.05) risk factors for LVDD, whereas LAVI > 37 ml/m^2^, lateral e’ < 8 cm/s, E/lateral e’ > 13, and SOFA > 5 were significant (*P* < 0.05) independent risk factors for LVDD.

LVDD refers to the limitation of left ventricular active relaxation and passive filling capacity, which is an important diagnostic factor for diastolic heart failure and is closely related to increased incidences of diastolic heart failure and mortality. The prevalence of LVDD in patients with severe sepsis and septic shock has been reported to range 20%–57% [9, 23], and this high heterogeneity in the prevalence may be caused by different definitions of LVDD, different timing of echocardiographic evaluation in the sepsis course, and varied clinical settings (septic shock vs. severe sepsis). Research shows that LVDD is not static, but a dynamic phenomenon [24]. The decrease of left ventricular diastolic function suggests a poor prognosis; conversely, improving left ventricular diastolic function can increase survival [25]. Therefore, early detection of the influencing factors for LVDD and timely intervention can improve its clinical prognosis.

Studies have shown that the occurrence of LVDD is related to many factors such as clinical factors, cardiac structural parameters and circulating biomarkers [19, 20, 26, 27]. Among different cardiovascular diseases, age, hypertension, diabetes, coronary atherosclerotic heart disease (CHD), obesity and LAVI are risk factors for LVDD, with age as the strongest independent risk factor affecting LVDD [28, 29]. The study in Framingham healthy population in the United States showed that the risk of LVDD increased by 3.6 times with an increase of every 10 years of age [30]. In addition, plasma BNP and N-terminal pro-BNP level are closely related to the occurrence and severity of LVDD [20, 31, 32]. Grewal et al. [20] found that the combined model of age, gender, body mass index, diabetes, hypertension, CHD, atrial fibrillation, LAVI and plasma BNP was of high predictive values for moderate and severe LVDD in 181 patients with diastolic heart failure. Among them, plasma BNP > 100 ng/L or N-terminal pro-BNP > 600 ng/L and diabetes history were independent risk factors for predicting LVDD. Mak et al. [32] defined E / e ‘ > 15 as increased left ventricular end-diastolic pressure (LVEDP) and E/e’ < 8 as normal LVEDP, and they found that the plasma BNP concentration in E/e ‘ > 15 group was (463 ± 80) ng/L, which was significantly higher than that in 8 < E/e’ < 15 group [(122 ± 24) ng/l] and E/e ‘ < 8 Group [(97 ± 27) ng / l]. Using plasma BNP > 173 ng / L as the cut-off point to predict E/e‘ > 15, the sensitivity was 88% and the specificity was 83%. It has also been reported [33] that in 58 severe ICU patients with normal left ventricular systolic function who required mechanical ventilation, age, serum creatinine, sepsis, positive inotropic agents and SOFA scores are the independent risk factors for E/e’. When LVDD was defined by e’ ≤ 8 cm/s or / and E/e’ ≥ 13 cm / s, plasma N-terminal pro-BNP > 947 ng / l had a sensitivity of 73% and a specificity of 70%.

In the study by Landesberg et al. [8] including 262 patients with severe sepsis and septic shock, decreased e’ was significantly associated with age and essential diseases such as hypertension, diabetes and CHD. This suggests a pathological basis for an increased incidence of LVDD in patients with increased age and those concomitant with hypertension, diabetes and CHD. Sepsis further promotes the release of inflammatory factors, myocardial Ca^2+^ overload, nitric oxide release and myocardial microcirculation disorder, resulting in LVDD [34]. This also suggests that the severity of circulatory failure in patients with septic shock is closely related to LVDD. When the body is in a state of severe hypotension, reduced myocardial perfusion can lead to mitochondrial dysfunction and further affect the active relaxation ability of left ventricular myocardium.

At present, it is still controversial as for whether LVDD predicts the risk of death or poor prognosis in patients with sepsis. Landesberg et al. [8] reported that compared with patients with normal left ventricular diastolic function, patients with severe sepsis or septic shock complicated with LVDD had a sixfold increased risk of death. Similarly, Sturgess et al. [9] studied a small group of patients with septic shock and found that E/e’ in the death group was significantly higher than that in the survival group. After further adjusting APACHE II score, cardiovascular disease, fluid balance and other related risk factors, E/e’ was an independent predictor of in-hospital death in patients with septic shock. In addition to the traditional risk factors such as age, blood lactic acid and APACHE II score, our study showed that plasma BNP and E / lateral e ' were independent risk factors for LVDD in patients with septic shock. However, Pulido et al. [35] did not find that LVDD was associated with an increased risk of death at 30 days and 1 year in 106 patients with severe sepsis or septic shock. A recent meta-analysis of 636 patients with sepsis included in 7 studies showed that the incidence of LVDD in patients with sepsis was 48%, LVDD was significantly correlated with poor prognosis, and left ventricular systolic dysfunction was not correlated with poor prognosis [13]. In a meta-analysis of patients with severe sepsis [13], LVDD was associated with mortality at the longest follow-up (relative risk 1.82, 95% confidence interval 1.12–2.97, *P* < 0.05). Therefore, early identification of sepsis complicated with LVDD is particularly important, especially for patients with fluid reactivity. In addition to active anti-infection treatment, early and effective fluid resuscitation can improve myocardial diastolic function and reduce mortality.

The presence of LVDD in patients with severe sepsis and septic shock has some significant clinical implications. The use of beta-blockade and noradrenergic sparing agents (vasopressin) in these patients may improve the prognosis and outcome because these agents may lower the heart rate to improve the diastolic function [36, 37]. This is critical because the suggested elevated efficiency of diastolic filling in tachycardia is restricted in sepsis [38]. LVDD has been reported to have a significant correlation with raised troponins in severe sepsis [39], and this correlation may reflect impaired myocardial relaxation from myocardial oxygen supply demand imbalance, possibly resulting from excessive catecholamines, tachycardia and/or microvascular dysfunction. This potential ischemia may cause diastolic dysfunction, making it imperative that myocardial work and oxygen demand be decreased.

Sepsis may induce liver injury which is recognized as a powerful independent predictor of mortality in the intensive care unit [40]. During systemic infection, the liver adjusts immune defense through bacterial elimination, manufacture of cytokines and acute-phase proteins, and adaptation to infection. However, the liver is also a target of sepsis-induced injury, including cholestasis, hypoxic hepatitis and drug-induced liver injury in critically ill patients [41]. Elevated levels of inflammatory cytokines, impaired bacterial clearance, and metabolic products can lead to gut microbiota dysbiosis and disruption of the intestinal mucosal barrier, resulting in systemic inflammatory response and acute liver injury [40]. In our study, patients with septic shock and LVDD had significantly (*P* < 0.05) increased BUN, creatinine, and triglyceride, which may suggest severe hepatic function damage in these patients. The aspartate aminotransferase and alanine aminotransferase were also increased, but did not reach the significance level, and if more patients were included, these aminotransferases may be significantly increased in septic patients with LVDD compared with those without LVDD.

Some limitations existed in this study, including the retrospective and one-center study nature, a small cohort of patients, and Chinese patients enrolled only, which may all affect the generalization of the outcome. Future studies will have to resolve all these issues for better outcomes.

In conclusion, patients with septic shock and combined LVDD are significantly different from those without LVDD in serum creatinine, APACHE II score, serum glucose, triglyceride, BUN, FT4, LAVI, mitral E, average e’, E/average e’, septal e’, septal e’/septal s’, E/septal e’, lateral s’, lateral e’, and E/lateral e’. LAVI > 37 mL/m^2^, septal e’ < 7 cm/s, septal e’/septal s’ < 0.8, E/septal e’ > 15, lateral e’ < 8 cm/s, E/lateral e’ > 13, lateral s’ < 10, average e’ > 10, E/average e’, APACHE II score > 16, SOFA > 5, BUN > 12 mmol/L, serum creatinine > 146* μmol/L*, serum glucose > 8 mmol/L, and triglyceride > 1.8 mmol/L are significant (*P* < 0.05) risk factors for LVDD, whereas LAVI > 37 ml/m^2^, lateral e’ < 8 cm/s, E/lateral e’ > 13, and SOFA > 5 were significant (*P* < 0.05) independent risk factors for LVDD.

## Data Availability

Data can be obtained from the corresponding author on reasonable requirements.
